# Engagement, not personal characteristics, was associated with the seriousness of regulatory adjudication decisions about physicians: a cross-sectional study

**DOI:** 10.1186/s12916-019-1451-1

**Published:** 2019-11-27

**Authors:** Javier A. Caballero, Steve P. Brown

**Affiliations:** 1 General Medical Council, 350 Euston Rd, London, NW1 3JN UK; 2 University of Manchester, Faculty of Biology, Medicine and Health, Oxford Rd, Manchester, M13 9PT UK; 3 The University of Sheffield, Dept of Psychology, Western Bank, Sheffield, S10 2TN UK

**Keywords:** Age, Sex, Race, Place of qualification, Ability to practise, Medical error, Professional regulation, Engagement with formal process, Regulatory sanctions

## Abstract

**Background:**

Outcomes of processes questioning a physician’s ability to practise —e.g. disciplinary or regulatory— may strongly impact their career and provided care. However, it is unclear what factors relate systematically to such outcomes.

**Methods:**

In this cross-sectional study, we investigate this via multivariate, step-wise, statistical modelling of all 1049 physicians referred for regulatory adjudication at the UK medical tribunal, from June 2012 to May 2017, within a population of 310,659. In order of increasing seriousness, outcomes were: no impairment (of ability to practise), impairment, suspension (of right to practise), or erasure (its loss). This gave adjusted odds ratios (OR) for: age, race, sex, whether physicians first qualified domestically or internationally, area of practice (e.g. GP, specialist), source of initial referral, allegation type, whether physicians attended their outcome hearing, and whether they were legally represented for it.

**Results:**

There was no systematic association between the seriousness of outcomes and the age, race, sex, domestic/international qualification, or the area of practice of physicians (ORs *p*≥0.05), except for specialists who tended to receive outcomes milder than suspension or erasure. Crucially, an apparent relationship of outcomes to age (Kruskal-Wallis, *p*=0.009) or domestic/international qualification (*χ*^2^,*p*=0.014) disappeared once controlling for hearing attendance (ORs *p*≥0.05). Both non-attendance and lack of legal representation were consistently related to more serious outcomes (ORs [95% confidence intervals], 5.28 [3.89, 7.18] and 1.87 [1.34, 2.60], respectively, *p*<0.001).

**Conclusions:**

All else equal, personal characteristics or first qualification place were unrelated to the seriousness of regulatory outcomes in the UK. Instead, engagement (attendance and legal representation), allegation type, and referral source were importantly associated to outcomes. All this may generalize to other countries and professions.

## Background

Are regulatory sanctions related to personal characteristics of physicians such as age, race, or sex? This question is of wide interest globally [1–7], especially for the domains of policy-making, education, employment, and liability insurance; because such characteristics, and whether physicians qualified domestically or internationally, figure centrally in live migration and equality issues [8–13].

The proneness of physicians (i.e. medical practitioners) to complaints, claims, disciplinary actions, or regulatory outcomes is traditionally attributed to one or more of their personal characteristics, area of practice, or place of first qualification (see, e.g. [1, 14–20]). Only a minority of comparable studies incorporate what the case was allegedly about (∼1/4 of found studies, e.g. [3, 6, 21–24]) or the source of initial referral (∼1/20 of studies, i.e. [17, 25]). Here we statistically analysed the sanction patterns of all physicians reaching a regulatory adjudication stage, within a whole national population of physicians, controlling for all these variables simultaneously. Further, motivated by an earlier small-scale exploration [26], this is the first study to control for variables related to the engagement of physicians with a process assessing issues alleged about their practice — here attendance at hearings and legal representation; thus revealing them as critical correlates of outcomes.

We show that variables related to engagement with regulatory processes and case-type may need to be more of a focus than personal characteristics. In particular, differences in engagement by particular groups, such as older physicians and international graduates, must be considered to avoid inaccurate conclusions. We hypothesize that similar patterns may underlie outcome differences in systems around the world dealing with complaints, legal claims, regulatory, or disciplinary actions.

## Methods

Anyone may submit an *initial referral* about a physician in the UK alleging an impairment in ability to practise (*practise impairment* for short), to the General Medical Council (GMC). Cases may be either resolved by the GMC or referred to the Medical Practitioners Tribunal Service (MPTS). A regulatory adjudication *outcome* is determined there for each case. Only the MPTS can determine the most serious outcomes of *suspension* of the right to practise or *erasure* (its loss). Here, *referral for adjudication* will mean a referral for *hearing* by a tribunal at the MPTS. At any stage, it may be decided that no further action is required, and that is the endpoint of the great majority of initial referrals [27]. Though warranting further research, analysing what factors relate to every interim regulatory stage, from initial referral to referral for adjudication, is beyond the scope of this study.

### Data sourcing and preparation

We sourced the regulatory record data from national-level databases maintained by the GMC and MPTS. For general context, we analysed data on the whole population of physicians registered in the UK in the initial-referral period from 01 Jun 2012 (MPTS inception) to 31 Dec 2016 (310,659 in total). We focused our main analyses on the cases of all physicians referred for adjudication, which received a hearing outcome by 31 May 2017. A total of 1236 MPTS hearings concluded in the period, involving 1049 distinct physicians — our unit of analysis. All of our statistical methods assumed comparability and independence across observations. Therefore, where a physician was involved in more than one hearing in the period, we used only the first appearance.

We consolidated decisions by the MPTS, in increasing order of seriousness, as: no impairment (to practise), impairment, suspension, or erasure (Table 1). Physicians’ attendance at a hearing was recorded throughout and legal representation for it since 2015. For seven physicians, attendance was unknown. For simplicity, we consolidated these ‘unknown’ attendance instances into the ‘yes’ category, since the distribution of outcomes for instances with attendance ‘unknown’ was more similar to that of ‘yes’ than it was to that of ‘no’. As a sensitivity check, we built two sets of 15 models each (as below) differing only on whether we used this consolidation (as finally reported) or not, and verified that the same conclusions would be drawn from either approach.

**Table 1:** Physicians referred for adjudication by their characteristics

Variable	Categories	No.	(%)
Allegation	Misconduct	344	(32.8)
	Performance	312	(29.7)
	Conviction	210	(20.0)
	Health	126	(12.0)
	Other	57	(5.4)
Area of practice	GP	307	(29.3)
	Specialist	263	(25.1)
	Neither (not in training)	396	(37.8)
	Neither (in training)	83	(7.9)
Attended	Yes	695	(66.3)
	No	354	(33.7)
Legally represented	Yes	257	(24.5)
	No	233	(22.2)
	Unknown	559	(53.3)
Licensed	Yes	1 021	(97.3)
	No	28	(2.7)
(Decision) outcome	No impairment	241	(23.0)
	Impairment	116	(11.1)
	Suspension	384	(36.6)
	Erasure	308	(29.4)
Outcome year	2012	120	(11.4)
	2013	210	(20.0)
	2014	211	(20.1)
	2015	225	(21.4)
	2016	213	(20.3)
	2017	70	(6.7)
PMQ region	ROW	487	(46.4)
	UK	398	(37.9)
	EEA	164	(15.6)
Race	BME	463	(44.1)
	White	321	(30.6)
	Not recorded	265	(25.3)
Sex	Male	895	(85.3)
	Female	154	(14.7)
Source (of initial referral)	Employer	411	(39.2)
	(Member of the) public	134	(12.8)
	Another physician	104	(9.9)
	Self-referral	79	(7.5)
	Police	69	(6.6)
	Regulator	58	(5.5)
	Other	194	(18.5)
	Total	1 049	(100.0)

We coded the allegation type (shortened to *allegation* ahead; not the allegation’s seriousness) as being about: misconduct (honesty or fairness, excluding probity or criminality), conviction (probity or criminality), (physician’s) health, performance (clinical, professional, communication, or respect), or other. Due to the small number of *outcome years*, we treated this variable as categorical throughout.

The race of a physician was self-reported. When recorded as ‘Asian’, ‘black’, ‘mixed’, or ‘other’, we consolidated race as *black and minority ethnic* (BME). When there was no data or physicians declined to report their race, this was consolidated as *not recorded*. Physicians in our data received their first or primary medical qualification (PMQ) in one of 64 countries, which we consolidated into world regions (*PMQ regions*). When their PMQ was in the UK, we counted physicians within the *UK* region. In the *EEA* region, we grouped physicians gaining a PMQ in any state currently within the European Economic Area, excepting the UK. We counted physicians obtaining a PMQ elsewhere as *rest of the world* (ROW) graduates. Our consolidation of race and PMQ region was driven by the distribution of our data, to render reasonably numerically balanced categories, and by preserving statistical power and maximum relevance to policy-makers.

Age, *licensed* status (holding a right to practise in the UK), and *area of practice* were those at the time of the initial referral to the GMC. In *area of practice*, we coded whether a physician had qualified as a GP, specialist, neither but was undergoing training towards either, or neither and was not undergoing training. We consolidated this variable out of the physician’s registration and training records. We counted the few physicians that were both GP and specialist as specialists.

### Statistical analysis

We conducted our analyses via bespoke scripts, coded in Stata (13.0), Python (3.6), and R (3.3.3). Throughout, we considered an association present if the relevant *p*<0.05.

#### Statistical testing

We conducted exhaustive statistical testing for association between all possible variable pairs, on their own. Of these tests, 42% gave a *p*≥0.05, suggesting that our sample size was not so large that testing would render significance even with negligible effect sizes. When both variables in a pair were categorical, we used chi-squared (*χ*^2^) or Fisher’s exact tests. Given the sensitivity of the research topic, we base our conclusions exclusively on results from non-parametric tests, rather than parametric ones. In particular, visual inspection of category-grouped age histograms, against best fitting Gaussian distributions, often suggested non-Gaussianity and that data transformation was unlikely to solve it. Therefore, we favoured results from the Kruskal-Wallis *H* test for age groupings, over those of the analogous analysis of variance.

#### Modelling

We sought to describe the systematic associations between characteristics of physicians and their cases, and adjudication outcomes. Outcomes were multi-class (non-binary) and were naturally ordered in seriousness. Therefore, we modelled them with partial proportional odds models — a generalization of the ordered logistic model — via the Stata *gologit2* user-written module [28]. We allowed gologit2 to automatically keep *parallel lines*— or equal odds ratios (OR) for all comparisons in a model — for every feature where the corresponding *p*≥0.05 in a Wald test for this assumption.

In a step-wise fashion, we produced 15 interim models (Table 2). We incorporated independent variables one by one in order of high to low association to outcomes, as suggested by *p*-values from statistical testing (models 1 to 11). We kept them if they improved the parsimony of the resulting model, measured by a substantial reduction in AIC. In model 12, we brought back in age, race, sex, and domestic/international qualification, as they were of central interest. A *p*=0.82 in a global Wald *χ*^2^ test indicated that model 12 did not violate the parallel-line assumption. Lastly, we fitted three confirmatory models (13 to 15) to observe the effect of not controlling for either attendance, legal representation, or both, correspondingly. For simplicity and interpretability, we refrained from including interactions between independent variables or non-linear transformations of them.

**Table 2:** Step-wise model building sequence (model No.)

Model No.	Attended	Legally represented	Allegation	Source	Area of practice	Age	PMQ region	Outcome year	Sex	Licensed	Race	AIC	AIC change
1	∙											2534.7	
2	∙	∙										2522.0	−12.7
3	∙	∙	∙									2458.1	−63.9
4	∙	∙	∙	∙								2449.0	−9.1
5	∙	∙	∙	∙	∙							2441.1	−7.9
6	∙	∙	∙	∙	∙	∙						2440.6	−0.5
7	∙	∙	∙	∙	∙		∙					2440.3	−0.3
8	∙	∙	∙	∙	∙			∙				2448.0	7.7
9	∙	∙	∙	∙	∙				∙			2441.2	−6.7
10	∙	∙	∙	∙	∙					∙		2441.4	0.2
11	∙	∙	∙	∙	∙						∙	2444.8	3.4
12	∙	∙	∙	∙	∙	∙	∙		∙		∙	2445.1	0.2
13		∙	∙	∙	∙	∙	∙		∙		∙	2566.6	121.5
14	∙		∙	∙	∙	∙	∙		∙		∙	2458.3	−108.2
15			∙	∙	∙	∙	∙		∙		∙	2639.3	181.0

Marker: variable included in interim model. *AIC* Akaike information criterion, *AIC change* AIC per row minus that of the previous row

## Results

From the MPTS inception in June 2012, to December 2016 inclusive, the GMC received initial referrals for 27,411 identified individual physicians. Of those, 1049 physicians received an adjudication outcome before June 2017 (descriptive statistics in Table 1). These were 0.34% of the 310,659 physicians holding a registration during the period.

### Engagement-related variables bore the strongest associations with the seriousness of outcomes

**Fig. 1:**
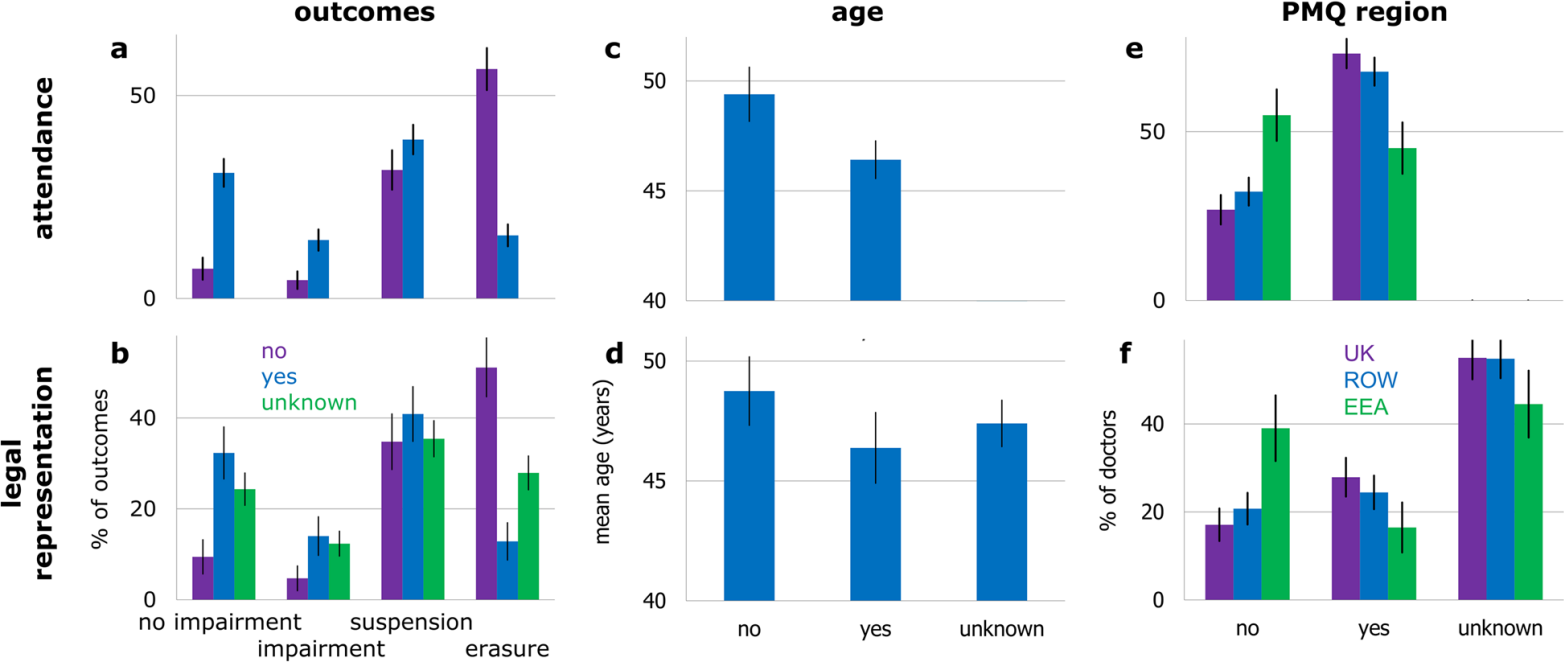
Relationship between engagement variables and: outcomes (**a**, **b**), age (**c**, **d**), and PMQ region (**e**, **f**). Top row: hearing attendance. Bottom row: legal representation. Black bars are Gaussian-approximated 95% confidence intervals (CI). See Table 1 for denominators

Only about half of the physicians for whom legal representation was known, obtained it (Table 1). Also, around two thirds of the physicians referred for adjudication attended their outcome hearing. Physicians that did not attend or obtain legal representation for their hearings tended to receive worse outcomes (Fig. 1a, b).

Physicians who attended had legal representation 77% of the times, whilst non-attendees had it 11% of the times (among physicians whose engagement we have data on). Thereby, attendance and representation were highly concurrent (*χ*^2^,*p*<0.001). In order, attendance and legal representation were the variables that bore the strongest bivariate association with outcomes (*χ*^2^,*p*<0.001). Controlling for confounds revealed that both not obtaining legal representation and non-attendance consistently related to more serious outcomes, though the average effect-size of non-attendance was much larger (Table 3); this where setting the ‘unknown’ category (a mix of latent ‘yes’ and ‘no’) as the base for representation purposefully resulted in representation only informing models when disambiguated.

**Table 3:** Results of the three simultaneous comparisons (grouped ORs and *p* values) in the final partial proportional odds model (model 12 in Table 2)

			All other *versus* no impairment		Suspension or erasure *versus*		Erasure *versus* all other	
					no impairment or impairment			
Variable	Base	Categories	OR [95% CI]	*p*	OR [95% CI]	*p*	OR [95% CI]	*p*
Age			1.01 [0.99 to 1.02]	0.29	-	-	-	-
Allegation	Other	Health	2.11 [0.95 to 4.72]	0.07	1.18 [0.61 to 2.30]	0.63	0.57 [0.29 to 1.14]	0.11
		Performance	0.58 [0.35 to 0.98]	0.04 ^*†*^	-	-	-	-
		Misconduct	0.89 [0.50 to 1.55]	0.67	1.20 [0.70 to 2.06]	0.51	1.02 [0.57 to 1.80]	0.96
		Conviction	1.17 [0.59 to 2.29]	0.66	1.70 [0.90 to 3.23]	0.10	2.06 [1.10 to 3.88]	0.03 ^*†*^
Area of practice	Neither (not in training)	Neither (in training)	0.79 [0.49 to 1.28]	0.34	-	-	-	-
		GP	0.87 [0.64 to 1.19]	0.39	-	-	-	-
		Specialist	0.71 [0.48 to 1.06]	0.09	0.51 [0.35 to 0.74]	<0.001^*†*^	0.85 [0.58 to 1.27]	0.43
Attended	Yes	No	5.28 [3.89 to 7.18]	<0.001^*†*^	-	-	-	-
Legally represented	Unknown	No	1.87 [1.34 to 2.60]	<0.001^*†*^	-	-	-	-
		Yes	0.85 [0.64 to 1.14]	0.28	-	-	-	-
PMQ region	UK	EEA	1.25 [0.86 to 1.81]	0.23	-	-	-	-
		ROW	1.33 [0.99 to 1.79]	0.06	-	-	-	-
Race	Not recorded	BME	1.01 [0.74 to 1.37]	0.96	-	-	-	-
		White	1.11 [0.80 to 1.55]	0.53	-	-	-	-
Sex	Female	Male	1.20 [0.86 to 1.68]	0.29	-	-	-	-
Source	Other	Public	0.46 [0.28 to 0.74]	<0.01 ^*†*^	0.46 [0.28 to 0.73]	<0.01 ^*†*^	0.82 [0.48 to 1.40]	0.46
		Employer	0.68 [0.49 to 0.96]	0.03 ^*†*^	-	-	-	-
		Another physician	0.60 [0.38 to 0.95]	0.03 ^*†*^	-	-	-	-
		Self-referral	1.60 [0.74 to 3.46]	0.23	1.25 [0.65 to 2.44]	0.50	0.38 [0.19 to 0.77]	<0.01 ^*†*^
		Police	0.91 [0.50 to 1.65]	0.76	-	-	-	-
		Regulator	0.78 [0.44 to 1.37]	0.38	-	-	-	-
Constant			2.09 [0.89 to 4.94]	0.09	1.07 [0.46 to 2.49]	0.88	0.15 [0.06 to 0.35]	<0.001 ^*†*^

Comparisons gave ORs for physicians receiving outcomes more serious than ‘no impairment’ rather than a ‘no impairment’ outcome; receiving ‘suspension’ or ‘erasure’ rather than ‘no impairment’ or ‘impairment’; or receiving erasure, instead of milder outcomes

^*†*^Significant at *α*=0.05. In this model, it was unnecessary to automatically relax the parallel-line assumption for most features. For clarity, ORs and *p* values for this majority are shown only at the leftmost comparison. They are equal for the rightmost two (dashes filled). See Table 1 for the data’s descriptive statistics

### Outcomes were systematically related to allegation and referral source

Allegation and referral source were intimately related (Fisher’s exact, *p*<0.001). For instance, between 2012 and 2016, the majority of complaints about physicians made to the GMC by the public were about performance, whereas the majority of those raised by the police were about conviction [27]. Both factors also appeared importantly related to the seriousness of regulatory GMC outcomes, before the creation of the MPTS [17]. This coincides with reports of allegation type relating to whether physicians received disciplinary actions in Australia [23] and the USA [3, 29].

Bivariate testing indicated an important association of either allegation or initial referral source, to outcomes (*χ*^2^,*p*<0.001). In turn, modelling revealed a particularly rich pattern of systematic associations (Table 3). To note, allegations of performance systematically related to less serious outcomes, whilst those of conviction related to erasure rather than milder outcomes. Also, self-referrals systematically associated to receiving outcomes less serious than erasure.

### Outcomes were associated with being a specialist but not with other areas of practice

The area of practice of physicians was on its own highly related to regulatory outcomes (*χ*^2^,*p*<0.001). However, after controlling for other confounds, this was no longer the case (Table 3). The only exception was for specialists, who tended to receive outcomes milder than suspension or erasure — matching prior findings [18] — compared to non-GP/specialists that were not in training.

### Outcomes were not associated with licensed status or year of outcome

The year of the hearing and whether physicians were licensed at the time of the initial referral were unrelated to outcomes, testing each relationship on its own (*χ*^2^ and Fisher’s exact, correspondingly, *p*≥0.05). This was confirmed by a *p*≥0.05 for relevant ORs in models including either variable (8 and 10 in Table 2), and by an increase in the AIC. In particular, the lack of association between hearing year and outcomes suggests that the MPTS determined outcomes consistently since its inception.

### Outcomes were not systematically related to age, race, sex, or domestic/international qualification

#### Age

**Fig. 2:**
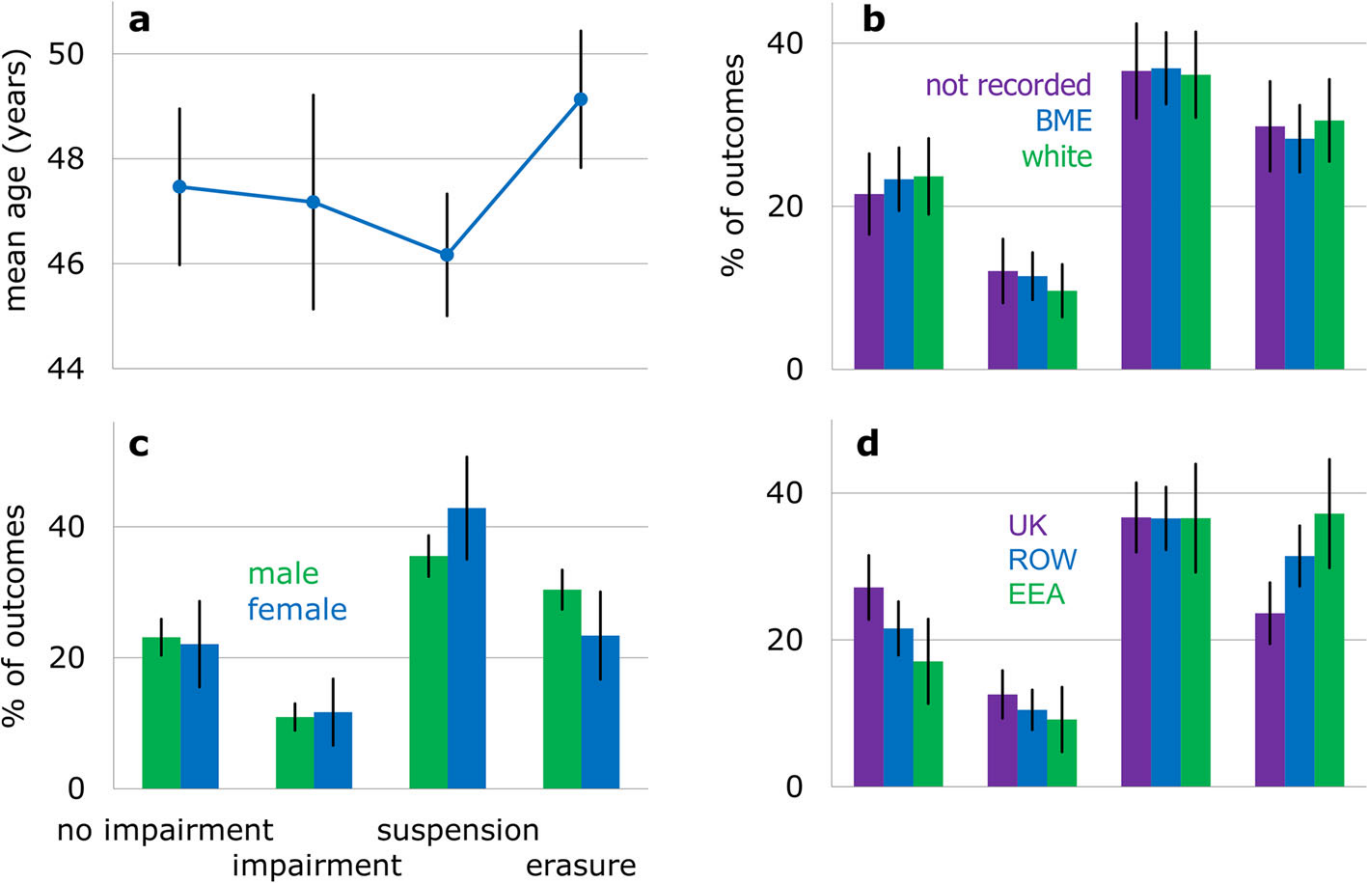
Outcomes by personal characteristics and PMQ region. **a** Age. **b** Race. **c** Sex. **d** PMQ region. Black lines are Gaussian-approximated 95% CIs. See Table 1 for denominators

The age of physicians referred for adjudication ranged from 23 to 82 years. On average, they were 5 years older (mean 47.4±11.7 std dev) than all registered physicians at the midpoint of the initial referral period (mean 42.1±13.2 std dev). Except for ‘erasure,’ the mean age of physicians was similar across outcomes (Fig. 2a).

Age on its own related to receiving different outcomes (Kruskal-Wallis, *p*=0.009). However, physicians that did not attend their hearing or were not legally represented were older on average (Fig. 1c, d), and the age-attendance or age-representation associations were important (Kruskal-Wallis, *p*<0.001 and *p*=0.038). Modelling indicated no evidence for age being systematically related to the seriousness of outcomes (Table 3). If, to confirm this, we were to not control for attendance, or for it and representation, it would appear that increasing age related to more serious outcomes (ORs with parallel lines in models 13 and 15 in Table 2, *p*<0.01). Not controlling for legal representation only would not change our conclusions (model 14; ORs *p*≥0.05). This resonates strongly with prior modelling reports not controlling for engagement variables and finding a relationship between physicians being older and the proneness to receive a legal claim [1, 30–33] or disciplinary action [34, 35].

#### Race

The proportion of BME physicians reaching an adjudication stage (44%, Table 1) was larger than that among registered BME physicians in the population in the period (31%). The case for physicians with ‘not recorded’ race was similar (25 versus 16%), implying that white race physicians were underrepresented. Similar representation patterns have been measured elsewhere in initial referrals [15, 36].

Despite this, we found no evidence for the race of physicians relating to their outcomes (Fig. 2b; *χ*^2^,*p*=0.95). Modelling verified that, all else equal, there was no evidence for the race group of a physician making a difference with respect to the seriousness of outcomes (Table 3). This coincided with prior findings in a disciplinary-action system [3] and in adjudication decisions at the GMC, before the MPTS inception [17]. Our results also contrast with findings that non-white physicians tended to receive a first disciplinary action earlier [25] or received more disciplinary actions [35].

#### Sex

Males made up 56% of the population in the period, yet made up 85% of physicians referred for adjudication (Table 1). Nonetheless, we found no bivariate evidence for an association between sex and outcomes (Fig. 2c; *χ*^2^,*p*=0.24). Modelling confirmed no evidence for a systematic association between sex and receiving more serious outcomes (Table 3).

This echoes, first, results from studies in several countries finding no relationship between a physician’s sex and the proneness to receive claims or disciplinary actions [5, 14, 37]. Second and most strongly, those of analyses finding no differences in the seriousness of disciplinary actions received between sexes [3, 38].

#### Domestic or international graduation

ROW and EEA graduates reached an adjudication stage disproportionately (46 and 16%, respectively, Table 1) compared to the physician population in the period (26 and 12%). UK graduates were thereby underrepresented (38 versus 62%). Nonetheless, this did not translate to systematic differences in outcomes.

There was a difference in prevalence of outcomes across PMQ regions (Fig. 2d; *χ*^2^,*p*=0.014). However, international graduates tended to not attend or obtain legal representation for their hearings more than domestic ones (Fig. 1e, f; *χ*^2^,*p*<0.001). Modelling then suggested no difference in the seriousness of outcomes between domestic and international graduates (Table 3). This agrees with reports worldwide on the reception of complaints [2, 4], legal claims [1, 2], or disciplinary actions [21, 25, 29]. Also, on the seriousness of disciplinary actions received, even without controlling for engagement [3]. As with age, we verified that this critically relied on us controlling for attendance (ORs in models 13 and 15, *p*<0.05), albeit not necessarily for legal representation (ORs in model 14, *p*≥0.05). This may explain why prior similar studies — that did not control for engagement variables whilst acknowledging possible remaining confounds [17, 18] — unknowingly found that international graduates were more prone to receive serious regulatory outcomes by the GMC (before MPTS inception).

## Discussion

We asked whether the seriousness of regulatory outcomes related to personal characteristics or to those of the case. In contrast to long-held beliefs, we found no systematic evidence for association of the seriousness of regulatory adjudication decisions to the age, race, sex, or domestic/international first qualification of physicians. This was critically due to us newly controlling for how physicians engage with their regulatory processes, which related to outcomes the strongest.

### Strengths and limitations

As in every study of this kind, there remains the possibility of important latent confounds yet to be identified, incontrovertibly measured, and controlled for. However, the most important limitation of this study is that we analysed the population of a single country. Hence, our findings can only be fully generalized within the UK. Notwithstanding this, they can also generalize partially elsewhere as there is a clear analogy to make between UK regulatory processes, aimed at determining impairment to practise, and systems in other countries and professions. This includes systems giving disciplinary actions (e.g. in Australia, Canada, the USA), legal claims (worldwide), and possibly even systems aimed at appropriately compensating affected patients without attaching to their cases the identity of involved physicians (e.g. in Sweden [7]).

The main strength of our study is its design. Studies on referrals, complaints, or claims naturally examine the relationship between a set of variables and whether the physician was referred or not. However, sometimes, the analogous question is also asked about disciplinary actions or regulatory outcomes, aiming to conclude about all physicians in the population, referred or not. The problem of this is that such design does not separate the decisions made by initial referrers to start a formal process from the decisions converting initial referrals to actions or outcomes, made by public bodies; thereby strongly incorporating the confound of the motivations of the referrer’s decisions. Instead, decisions by public bodies are better studied separately in a design comparing only referred physicians across.

The above is crucial because it neatly explains differences between our findings and those in the literature. For instance, males or international graduates are often overrepresented worldwide in multiple systems dealing with complaints, legal claims, disciplinary outcomes, or regulatory sanctions [2, 3, 6, 14–18, 21, 23, 29, 31, 33–35, 37–40], though international graduates are sometimes not overrepresented [14, 17, 29]. Multivariate analyses also show systematic associations to either group getting more serious outcomes [1, 2, 4, 6, 18, 19, 25, 29, 31, 33–35, 39], though there is counter-evidence [1, 2, 4, 5, 14, 21, 25, 29, 37]. However, all these multivariate analyses compared physicians receiving a process to those receiving none, rather than comparing outcomes across referred physicians. Hence, they retained the confounds in the motivations of initial referrers. Studies that did otherwise — like ours — consistently found no differences [3, 38].

### Why do older and internationally graduated physicians engage less readily?

Among many possible reasons, it seems natural to speculate that older physicians may decide it is not worth engaging due to being closer to retirement. International graduates may possibly consider the alternative of returning to the country where they originally qualified. Some indication of the latter is in ROW graduates attending and being represented substantially more often than EEA ones (Fig. 1e, f). Arguably, it is simpler for EEA graduates to return to their country of PMQ, than it is for ROW ones.

Not attending and/or not getting legal representation may of course relate to physicians a priori assuming a serious hearing outcome. Attendance and legal representation were highly concurrent. Also, the allegation type related to both attendance and representation (*χ*^2^,*p*<0.001 and *p*=0.024, respectively), though source only related to attendance (*χ*^2^,*p*=0.02 and *p*=0.33, correspondingly). All this may support the hypothesis that the outcomes expected by physicians play a role in their decision to attend or obtain legal representation. Further research is worth pursuing to clarify this.

### International implications

Physicians worldwide currently appear equally capable to practise, regardless of their place of graduation, though it has been raised that internationally graduated physicians may have fewer resources, connections, less confidence, or support [17]. There is also evidence that they often take positions that are less attractive, have higher workloads, serve more deprived areas, and are insufficiently resourced [13]. Furthermore, when directly compared, quality of care appears no different to that of domestic graduates in multiple countries [41–44].

In the UK — that relies heavily on the immigration of physicians qualified elsewhere [13, 45] — we found no differences in seriousness of regulatory outcomes across qualification places. Globally, our findings call for future studies to control for case-specific and engagement factors to determine their bearing on comparable formal processes.

## Conclusions

All else being equal, we found no evidence for associations between the seriousness of regulatory adjudication outcomes and the age, race, sex, area of practice of referred physicians, or whether they qualified domestically or internationally. The only exception was a tendency for specialists to receive outcomes less serious than suspension or erasure, compared to non-GP/specialists that were not in training.

We also showed that engagement — in the sense of attendance at hearings and legal representation — had the strongest relationship with outcomes, followed by case characteristics — allegation type and referral source.

## Data Availability

Due to data protection regulations, the source data cannot be supplied. Anonymized summaries of it are supplied in this manuscript.

## Abbreviations

AIC: Akaike information criterion
BME: Black and minority ethnic
CI: Confidence interval
EEA: European Economic Area
GMC: General Medical Council
GP: General practitioner
MPTS: Medical Practitioners Tribunal Service
OR: Odds ratio
PMQ: Primary medical qualification
ROW: Rest of the world
std dev: Standard deviation
UK: United Kingdom
USA: United States of America
